# Global protein interactome exploration through mining genome-scale data in Arabidopsis thaliana

**DOI:** 10.1186/1471-2164-11-S2-S2

**Published:** 2010-11-02

**Authors:** Feng Xu, Guang Li, Chen Zhao, Yuhua Li, Peng Li, Jian Cui, Youping Deng, Tieliu Shi

**Affiliations:** 1 College of Life Sciences, the Northeast Forestry University, Harbin, Heilongjiang 150040, China; 2 The Center for Bioinformatics and Computational Biology, and The Institute of Biomedical Sciences, School of Life Science, East China Normal University, 500 Dongchuan Road, Shanghai 200241, China; 3 Shanghai Information Center for Life Sciences, Chinese Academy of Sciences, Shanghai, China, 200031; 4 Rush Cancer Center, Rush University Medical Center, Chicago, IL 60612, USA; 5 Department of Biological Sciences, University of Southern Mississippi, Hattiesburg, MS-39406, USA; 6 Daqing Institute of Biotechnology, Northeast Forestry University, Daqing, Heilongjiang 163316, China

## Abstract

**Background:**

Many essential cellular processes, such as cellular metabolism, transport, cellular metabolism and most regulatory mechanisms, rely on physical interactions between proteins. Genome-wide protein interactome networks of yeast, human and several other animal organisms have already been established, but this kind of network reminds to be established in the field of plant.

**Results:**

We first predicted the protein protein interaction in *Arabidopsis thaliana* with methods, including ortholog, SSBP, gene fusion, gene neighbor, phylogenetic profile, coexpression, protein domain, and used Naïve Bayesian approach next to integrate the results of these methods and text mining data to build a genome-wide protein interactome network. Furthermore, we adopted the data of GO enrichment analysis, pathway, published literature to validate our network, the confirmation of our network shows the feasibility of using our network to predict protein function and other usage.

**Conclusions:**

Our interactome is a comprehensive genome-wide network in the organism plant *Arabidopsis thaliana,* and provides a rich resource for researchers in related field to study the protein function, molecular interaction and potential mechanism under different conditions.

## Background

A protein in an organism does not fulfill its function independently. The complicated cellular functions of an organism frequently rely on physical interactions between proteins. Over the past decade, various new experimental methods have been developed to quantify the interaction between proteins. Some of them, like AFM, FRET and BREF, can only be applied on the small scale protein-protein interaction detection, while others, such as Yeast Two Hybrid, Affinity Purification and Protein Arrays, are suitable for high throughput purpose. High throughput technologies, like yeast two-hybrid system (Y2H) and affinity purification, have been applied for genome-wide detection of protein protein intearction in several species, including *Homo Sapiens*[1-4], *Drosophila Melanogaster*[5,6], *Saccharomyces Cerevisiae,*[7-9]and *Caenorhabditis elegans*[10].

In *Homo Sapien,* Wanker and his colleagues have constructed the protein-protein interaction network with 3186 protein pairs containing 1705 proteins, while Vidal et. al. have built a protein-protein interaction network with the same platform. However, only a few small overlapping pairs are detected between the two protein interaction datasets. Same phenomenon also is observed during the process of building protein protein interaction network in *Drosophila Melanogaster.* Giot et al have built an interactome of *Drosophila Melanogaster* containing 4,679 interactions between 4,780 proteins. Later on, another group reported a protein interaction map with 2338 interactions between 1727 proteins using a high-throughput yeast two hybrid system. Surprisingly, only 24 interactive pairs are overlapped between the two datasets. The same results emerge in *Saccharomyces Cerevisiae* also. Among three different PPI datasets generated by three independent research groups with 4,475, 72,690, and 48,751 PPI pairs, respectively, maximum overlaps are below 9% between each two interactomes. The first two interactomes are generated with the same technology - yeast two hybrid system, and the last one is done with Affinity Purification-MS method. These previous independent results show a low consistence between different research groups. Analyses of the differences in the datasets and methods above indicate that the high throughput experimental methods have condition-specific and method-specific characteristics, which result in the little overlap even with the same experiment platform in the same species. Thus each of the high throughput experiment methods has its bias in detecting protein-protein interaction and alternative strategies should be adopted to enhance the accuracy and comprehensiveness of the genome-wide protein interaction maps. However, the high-cost, low coverage, and labor intensive of those experimental methods limit their applications.

In order to overcome the drawbacks and the limitation of the experimental approaches and expand the coverage of protein protein interaction network, various bioinformatics approaches have been developed during past decade. For example, new bioinformatics methods, based on the gene expression profile, the evolutionary relationship and genome contexts, are introduced in the field of genome-wide protein-protein interaction network construction, those methods include ortholog[4,11-13], microarray gene expression profiles[4,7,12-15], gene fusion[15], gene neighbour[15], phylogenetic profile[16], domain[4,12,13] and SSBP[4,12,13]. Up to now, the interactomes of several model organisms, such as *Homo Sapiens, Saccharomyces Cerevisiae, Drosophila Melanogaster, Caenorhabditis Elegans, Plasmodium Falciparum* and *Arabidopsis Thaliana,* have been already built on the basis of certain bioinformatics methods. These available interactomes have been constructed by combining different high-throughput data with bioinformatics predicted results. For example, the integrated protein-protein interaction network of *Saccharomyces Cerevisiae* was derived through the integration of the Y2H data, protein complex and microarray gene expression profiles with the Bayesian probability model [7]. The protein interactome of Human was built through probabilistic integration of the results generated from protein-protein interactions in several model organisms, protein domain assignments, gene expression measurements and biological function annotations [4]. The two protein-protein interaction networks in *Arabidopsis thaliana* were constructed based on the data of ortholog and collected transcriptome data, or shared annotation, domain interaction, and co-localization [13,17]. The *Plasmodium falciparum* protein interactome [15] was established on the basis of the methods including gene fusion, phylogenetic profile and gene expression data. All of the above interactomes except Arabidopsis are generated with Bayesian integration approach. As different bioinformatics methods predict the protein-protein interactions based on different types of data, each one also has its own inherent bias and limited coverage [18]. To systematically integrate those various data and make the results consistent to increase the prediction coverage and accuracy, various algorithms ,such as Voting, SVM, and Bayesian probabilistic model have been adopted for the purpose. Since Voting just uses a limited partition of data and SVM needs parameter estimation which might bring uncertainty into the prediction result, Bayesian probabilistic model has been widely applied in the integration process of establishing the genome-wide protein-protein interaction networks [4,15,19].

By assembling a collection of genomic, transcriptomic and proteomic data, we have successfully predicted genome-wide protein-protein interactions in *Arabidopsis thaliana* on the basis of model organism protein-protein interactions, protein domain assignments, *Arabidopsis thaliana* gene expression profiles, biological function annotations, and genomic inference methods. During the process, we have used carefully constructed high quality Golden Standard Positive (GSP) data and Golden Standard Negative (GSN) data to enhance the reliability of Bayesian probabilistic model and generated the protein-protein interaction network with a larger coverage. Next, we have carried out functional analysis on the basis of our *Arabidopsis thaliana* PPI network. We have also annotated protein function by using the simulated annealing method. In addition, we have deposited all our results and other integrated useful resources into AtPID [20]. The results of our analysis show the wide applicability of our network and demonstrate that our interactome is a valuable resource for biologist in plant field to investigate the protein function and explore the potential mechanism of various life processes.

## Results

During the process, we first defined a gold standard positive set (GSP) through text mining from published literature as well as data collection from IntAct, BIND and TAIR database. We generated a gold standard negative set (GSN) on the basis of the different sublocation information from GO. Data integration was mainly fulfilled by measuring individual accuracy in the context of overlap with the gold standards, and subsequently assigning them weights or scores according to the number of overlaps. The scores calculated here were then used to construct the main protein-protein interaction network of *Arabidopsis Thaliana.* After the construction of the network, we validated our network by using data from KEGG[21,22], AraCyc[23,24] and published literature.

### Gold standard sets

A gold standard positive (GSP) set of 4,129 distinct protein-protein interactions among 5,505 proteins came from the databases IntAct [25], BIND[26,27], TAIR [28,29] and text mining (see methods). Such GSP set was used to score the interaction network and to assign each predictive interaction pair with quantitative measurements. A gold standard negative set (GSN), within which each component of a pair has different cellular sublocation according to the Gene Ontology database, contains 196,855 protein pairs (see methods). Thirteen pairs of identical protein-pair, which appeared in both sets, were removed from both GSP and GSN.

### Network construction

**Figure 1:**
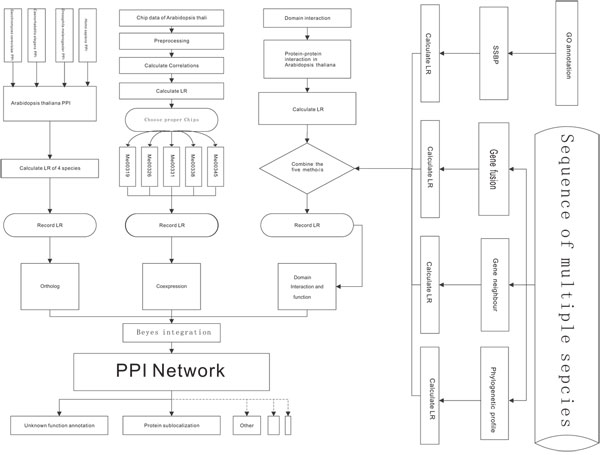
The flowchart of our interactome construction process 1. the LR score of each method is calculated first, we next combined the results LR of gene neighbor, gene fusion, phylogenetic profile and SSBP together and validated the PPI pair obtained by these four methods with protein domain method. Only the PPI that is predicted by one of the above four methods and is supported by domain could be integrated in our interactome. 2. We integrated the result of the newly combined methods with the results of ortholog and gene coexpression. The final LR score of our PPI network is the product of the LR scores of the three above methods.

We applied ortholog interactomes, expression data, shared biological function, enriched domain pairs, gene neighbor, gene fusion and phylogenetic profile combined with prior odds to predict protein-protein interactions. By applying Naïve Bayesian approach [30,31], we constructed our protein-protein interaction network in the following manner (Figure 1).

Since shared biological function represents the consistence of protein function more than real physical interactions and genome context methods (including gene neighbor, gene fusion and phylogenetic profile) imply functional linkage [16], the results of these methods reflect the functional linkages between the proteins rather than the true direct physical interaction. We validate the predictive result of these methods with domain interaction as domain interactions are the fundamental of function unit for physical interactions between proteins. Thus, only the protein-protein interaction pairs that are generated by the combination of genome context methods and shared biological function and, at the same time, are confirmed by inferred domain interaction are integrated into our network. The likelihood ratio (LR) of protein-protein interaction pair, based on the above five methods, is the maximum LR among the different genome context methods and shared biological function method. Next, we integrated the above results with Naïve Bayesian algorithm. The final LR of PPI pair within our network were generated from three parts, the LR according the above five method, the LR of expression data method and the LR of ortholog interactomes (See Methods).

After the construction of the network, we categorized PPI pairs into three groups according to LR score. Firstly, we applied the generally accepted Bayesian criterion, O_post_=1, to define the cutoff of high confident interactive pairs. Under this condition, the LR is calculated to be 559. There are 2505 interactions concerning 1361proteins in our high confident network. Secondly, As the interaction predictions based on ortholog are more reliable, among the LR generated from 4 different ortholog interactomes, *Caenorhabditis elegance* has the lowest LR with the score 92, we used 92 as the cutoff for the medium confident interactions in our network, the 16429 interactive pairs with 5363 proteins. Last, the interaction pairs having the LR score lower than 92 but being supported by at least one predictive method are collected in the low confident network. Thus, we use 92 and 559 as the likelihood ratio cutoff and separate our PPI network in low, middle and high confident groups.

### Network function annotation

The simulated annealing algorithm[32] was used to annotate the function-unknown proteins based on the concept of guilt-association in both high and medium confident PPI network. According to the annotation information in TAIR7, which was the latest edition of TAIR annotation information when we constructed the whole PPI network, there were 1,260 function unknown proteins in our high and medium confident PPI network. Through annotation process, all of these 1,260 proteins were annotated with 1,473 GO terms in 121,369 annotation pairs (each annotation pair is composed of a function unknown protein and its annotated GO term). 6,686 pairs of annotation had p-value with high significance (less than 0.0517), which can be treated as high confident annotation pairs. For example, protein AT5G02050, predicted as ATP-dependent helicase activity (GO: 0008026) by our method. is consistent with the TAIR 8 annotation mitochondrial glycoprotein family protein. The rest of the significant annotations are provided in additional file 1.

### Interaction conservation in other species

In order to verify the interactions in our network, the orthologs in *E.coliK12, S.pombe,* and *S. cerevisae* inferred from the Arabidopsis proteins in our high and medium confident networks are extracted from the Inparanoid database. Next, corresponding protein-protein interaction pairs from the individual organism were built in accordance with the protein-protein interaction in our interactome. Since the ortholog information in *S. cerevisae* was used to construct our PPI network, we first excluded the predicted PPI in *A. thaliana* inferred from the *S. cerevisae* orthologs in our network; we next reversely mapped the rest protein interaction pairs to *S. cerevisae.* Then, we search all of the new built pairs in IntAct databases. In this way, we obtained fifty conservative pairs in *S. cerevisae.* The result indicates that many of our predictive protein-protein interaction pairs are conserved in other organisms. For instance, our predictive interaction pair, AT2G33210 and AT2G39770, has corresponding interaction pair, NP_418567.1 and NP_416543.1, in *E.coliK12,* which has been confirmed by the pull down experiment [33]. In addition, the conservative protein-protein interaction pairs could also show biological function relationship between a protein and large biological complex. For example, the protein interaction pair YLL036C and YDL030W was deduced according to our predictive pair AT2G33340 and AT5G06160. In *S. cerevisae,* the protein YLL036C represents the splicing factor associated with the spliceosome and the protein YDL030W is a subunit of the SF3a splicing factor complex. The interaction pair confirms the interaction between splicing factor and the related spliceosome complex [34,35]. All of the inferred interaction pairs in Arabidopsis Thaliana and their corresponding PPI in other species are provided in Additional file 2.

### Consistence between pathway information and network

According to the data concerning pathway, we found that the proteins that participated in more pathways had the tendency to have a higher degree in our interactome, which is also supported by previous work[36]. We have used pathway related data of *Arabidopsis thaliana* from KEGG and AraCyc to check the consistence between pathway and our network for the validation of our interactome. There are totally 299 pathways in KEGG and AraCyc. Firstly, we counted the number of the pathways that a protein participates in. Among all the member proteins of these pathways there were 33 proteins that join in more than 10 pathways (Additional file 3 table 1) which can be regarded as hubs in the pathways. After the identification of these proteins, we located the hub proteins in our whole network, whose nodes have the average degree 373, comparing with the average degree 42 of all nodes in the whole network. Searching the degree of these joint components shows that the proteins related to more pathways have the tendency to have higher degrees.

**Table 1:** The related proteins and PPI pairs of four species from the DIP database. These data will be mapped to the genome of *Arabidopsis thaliana* based on ortholog relationship and be used to predict the PPI in the *Arabidopsis thaliana*.

Species	Protein numbers.	PPI numbers.
*Drosophila melanogaster*	7079	21013

*Saccharomyces cerevisiae*	4923	18331

*Caenorhabditis elegans*	2644	4038

*Homo sapiens*	1177	1734

**Figure 2:**
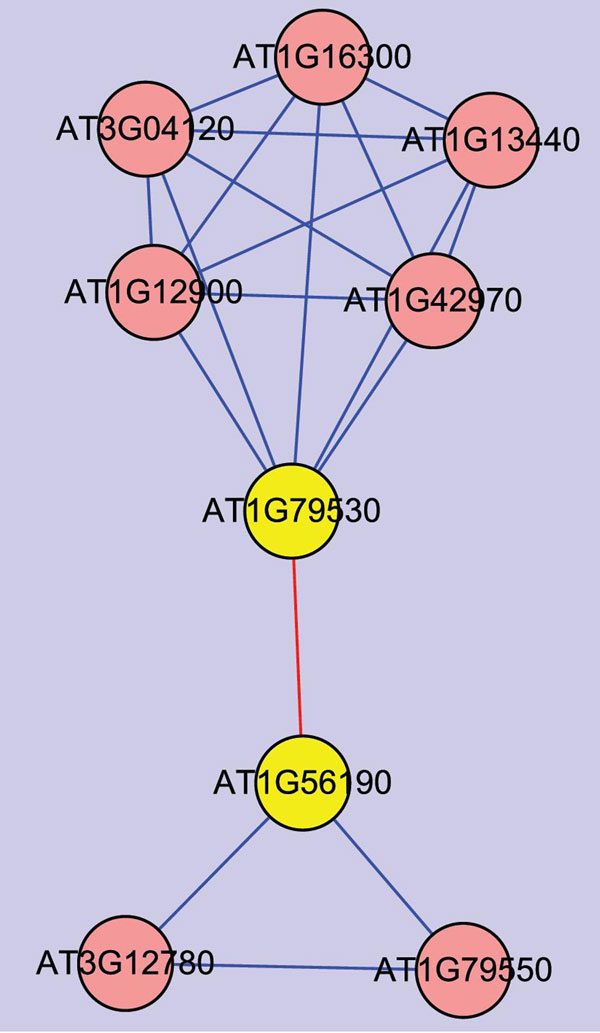
PPI network containing the interaction between At1G79530 and At1G56190 The PPI network is extracted from our medium confident bin. At1G79530 and At1G56190 (yellow node) has E.C code of 1.2.1.12 and 2.7.2.3, which have the same substrate according to the KEGG pathway ath00010, respectively. The red lines represent the interactions that show the consistence with the pathway information.

Besides, the components of our PPI pairs have the functional consistence. We collected all the components in the Glycolysis/Gluconeogenesis pathway (ath00010) from KEGG and used their neighbors in our high and medium confident network to build an ath00010 related PPI network. Figure 2 shows us that protein At1G79530 has an interaction with At1G56190. According to the information from KEGG, the EC code of At1G79530 and At1G56190 are 1.2.1.12 and 2.7.2.3, which represents glyceraldehyde-3-phosphate dehydrogenase and 3-phosphoglycerate kinase, respectively. In the KEGG pathway ath00010, the two proteins have the same substrate Glycerate-1,3P2. This functional consistence shows the reliability of our PPI network and PPI predictions.

Moreover, we have mapped all the pathways to our high and medium confident network. Almost all the metabolic pathways (299 pathways) have their key components in our network (Additional file 3). For example, the protein At2G44350, represents ATP citrate synthase, has a key function in the pathway Acetyl-CoA biosynthesis from citrate. In our network, the protein has interaction with several proteins to carry out the function, Those proteins include At1G53240 (malate dehydrogenase (NAD), mitochondrial), At3G28715 (H+-transporting two-sector ATPase, putative), At3G28710 (H+-transporting two-sector ATPase, putative), AT3G47520 (Encodes a protein with NAD-dependent malate dehydrogenase activity), AT3G09810 (isocitrate dehydrogenase, putative / NAD+ isocitrate dehydrogenase, putative). When mapping the At2G44350 gene to the orthologs in mouse and *S. pombe,* which we did not applied in our predictive process, we found that the related ortholog protein in both the organisms also interacted with malate dehydrogenase and other proteins concerning the function of energy metabolism. These functional consistencies related to mitochondrion and interactive conservation in energy metabolism across species could validate the value of our network in studying metabolic pathways or related function modules. Moreover, we have tried to extend the pathway with the help of our interactome. For example, the protein AT1G48790, which is annotated as ubiquitin-dependent protein, is not the component in the aerobic respiration alternative oxidase pathway. But in our network, AT1G48790 has interaction with AT1G16700 which is one of the components of the pathway represents NADH dehydrogenase (ubiquinone). In addition, AT1G48790 involves in the pathways such as NADH phosphorylation and dephosphorylation, oxidative phosphorylation, and aerobic respiration. it makes us suppose that the aerobic respiration alternative oxidase pathway might interact with other biological components or above pathways by means of the interaction between AT1G48790 and AT1G16700.

### Interaction between transcription factors

Cooperative transcriptional activations among transcription factors (TFs) are important to understand the mechanisms of complex transcriptional regulations in organisms. We used our protein-protein interaction data to infer the possible interaction between transcription factors. For example, we found interaction between AT1G56650, that involves in abscisic acid (ABA) signal transduction, and AT2G40220, that response to abscisic acid stimulus. Both of the TFs could be the downstream components of the ABA signaling pathway that participate into the regulation of target genes response to ABA signal. Therefore, these interactions between transcription factors may exposit certain regulatory mechanism of the transcription factors in the biological processes. Researchers could refer to AtPID to check the interaction partner of their interested transcription factors.

All our predicted PPI pair and the whole network is stored in *Arabidopsis thaliana* Protein Interactome Database(AtPID)[20], which is available at http://www.megabionet.org/atpid/. One can explore our interactome with the help of AtPID.

## Discussion

### The characteristics of our interactome

Because of the importance of protein-protein interaction, an interactome will be an invaluable resource for studying the protein functions, sublocations and protein protein interactions, and inferring the potential mechanisms of biology processes. Currently, two predictive interactomes of *Arabidopsis thaliana* are publically available. One of the interactomes is mainly based on the method of ortholog data combined with gene co-expression relationship, that contains 7,072 high and medium confidence protein-protein interaction pairs between 1,650 proteins [13], while another one is on the basis of gene expression, domain interaction, shared annotation, and co-localization including 224,206 potential interactions concerning 9,221 proteins[17]. Since each of the predictive methods has its own bias and coverage [18], the resulted datasets have just reflected certain aspects of the genome-wide protein protein interactions in *Arabidopsis thaliana.* Therefore, we constructed our interactome in a more comprehensive way by integrating the information concerning ortholog, coexpression, genome context, functional annotation, and protein domains with Bayes probabilistic approach. The number of the overlapped interactive pairs is 3,054, involving 1,444 proteins, between our high and medium confident interactome and Lee’s interactome. In contrast, our high and medium confident interactome only contained the same 791 consistent interactions between 635 proteins as Chen’s interactome. Similarly, the overlap between the previous two networks is just 338 interactions concerning 335 proteins. The differences between the datasets could result from the criterion used by different groups. Our domain based interactions came from two different approaches, one part of the interactions were constructed according to the directly domain interaction that were experiment validated, meanwhile, another part of the interactions were inferred from domain interaction based on the experimental confirmed protein interaction data. In contrast, the domain features in Chen’s methods were based on seven predictive methods come from Pfam system, thus the domain interaction criterion adapted in our methods is more stringent. Moreover, Lee’s and our group has adopted similar methods such as ortholog etc, that could contribute to higher overlapped interactive pairs between the two resulted interactomes. However, Lee’s group just uses ortholog to construct the interactome and the coverage of their interactome is much low than ours. Besides, during their analysis process, they simply evaluate the goodness and stability of a protein-protein interaction with a CV value without applying any powerful statistical integrative method. The differences among the three interactomes denote that each of the three datasets could be complementary to the others. In addition, more various methods could be applied in the field of protein protein interaction predictions to generate more comprehensive interactiome.

### The biological exploration of the whole network

The result of GO Enrichment Analysis shows the relative consistence between our network topology and biological ontology. We have used GO enrichment[37] to explore our large scale PPI network. The protein lists which are specially expressed in flower, leave, root, seed and silique[12] were extracted to do GO Enrichment Analysis with Amigo[37] first, and the result showed that these proteins were enriched in 48 GO terms. Next, we searched the neighbor of these tissue specific expressed proteins in our whole network. Then, we carried out the GO enrichment analysis on these neighbor proteins. Subsequently, we compared the results generated by the above two runs of GO Enrichment Analysis. The results show that direct biological relationships exist between the tissue specific proteins and their interaction partners in our interactome. The tissue specific proteins are enriched in many GO terms, one of which is GO: 0005739. This GO term lies in the eighth hierarchy of the GO hierarchical structure and its description is mitochondrion. The first level neighbors of proteins enriched in GO: 0005739 were also closely correlated in the same GO term (GO: 0005739) according to GO-enrichment analysis, with the descriptions such as purine ribonucleotide biosynthetic process, purine nucleotide metabolic process, ribonucleotide metabolic process and ATP biosynthetic process (Table 2). ATP is biosynthesized in mitochondrion and the *A* in ATP represents adenine that is a purine base. We performed similar analyses on the rest 47 GO terms, 26 of them showed direct biological relations (additional file 4). The direct biological consistence resulted from the GO enrichment analyses implied the biological reliability of our PPI network. With the consistence of the biological ontology, our interactome could be used to further elucidate the molecular aspects of proteome in *Arabidopsis thaliana* including the function annotation of unknown protein, sublocalization prediction of a certain protein, molecular experiment guidance.

**Table 2:** The result of GO enrichment analysis of proteins with GO: 0005739 mitochondrion with the GO term in the 8^th^ layer of GO hierarchical structure and their neighbors in our PPI network.

GO TERM	p-value	Description
9152	7.51E-14	purine ribonucleotide biosynthetic process

6163	1.09E-13	purine nucleotide metabolic process

9259	1.30E-13	ribonucleotide metabolic process

6754	7.21E-10	ATP biosynthetic process

We exert GO enrichment analysis on the proteins enriched in the GO: 0005739 and their interactive partners. The table contains the top GO terms these partners enriched in. The first column is the GO terms that the partners enriched in, the second column is the p-value of enrichment and the third is the functional description of the related GO terms.

### Functional unit in the interactome

The existence of interaction between two proteins suggests that they contribute to the same or similar biological processes. Many cellular processes and chemical events in organisms such as enzymatic reactions and dimerization involve protein-protein interactions, and function related proteins tend to form a function unit to carry out the specific biological process. We can unravel the complex interaction network to identify those function units and the potential new components in them with our interaction network.

Spliceosome are composed of snRNA, snRNP, splicing factors and other miscellaneous proteins. The interactions between these distinct proteins fulfill the function of removing introns from a transcribed pre-mRNA sequence. We did further exploration in our high and medium network. Firstly, we collected the proteins that are annotated as splicing factors, we next acquired their interaction partners and selected those proteins interacting with more than one protein annotated as splicing factor. In this way, we obtained a splicing functional unit containing 52 pairs PPI between 54 proteins. 80, 18, and 3 of these PPI pairs are conserved in yeast, human and *Drosophila,* respectively. For example, the interactive pair of AT3G55200 and AT1G07170 in our interactome has corresponding orthlog pairs of YML049C-YPR094W in yeast and ENSP00000305790- ENSP00000216252 in human. YML049C is annotated as a protein involved in pre-mRNA splicing; component of the pre-spliceosome, while YPR094W is annotated as a zinc cluster protein involving in pre-mRNA splicing and cycloheximide resistance. In addition, ENSP00000305790 is annotated as splicing factor 3B subunit 3 and ENSP00000216252 is annotated as splicing factor 3B-associated 14 kDa protein. All of these proteins commit the splicing activities. Thus, for the protein AT1G07170 annotated as a cellular component unknown protein in TAIR system, we can assign it to the splicing functional unit and it should have the biological function concerning splicing activities in *Arabidopsis thaliana.* Furthermore, among these PPI pairs, four of them are the interactions between 8 different splicing factor proteins. Moreover, 31 proteins that are not annotated as a splicing factor have interactions with at least two other splicing factors. Among these 31 proteins, eleven proteins, like AT2G18740, and AT3G53570, are annotated as small nuclear ribonucleoproteins, which are the components of spliceosome. These consistencies not only validate the composition of the spliceosome but also reflect the biological applications of our interactome. In the functional unit, other proteins that do not have direct relationships with spliceosome may correspond to the other miscellaneous proteins in spliceosome complex, these proteins could be used to infer the potential functions of the splicing factors in other biological processes. Therefore, our interactome offers value clues to explore the new functions for proteins and the potential mechanisms in the different biological conditions.

### Literature validation

**Figure 3:**
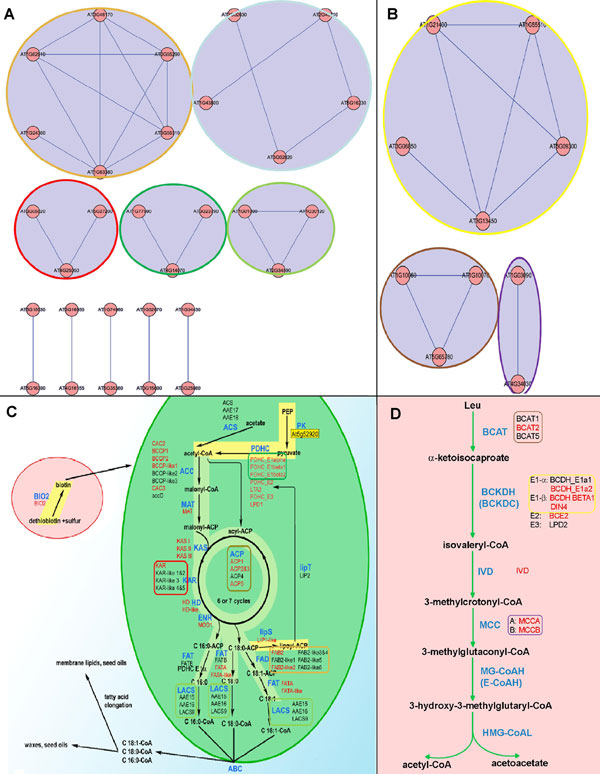
(A, B) PPI network and corresponding processes related to fatty acid biosynthesis and leucine catabolism. The component proteins of fatty acid biosynthesis and leucine catabolism in *Arabidopsis thaliana* are from a published paper [38]. A: The sub-networks related to fatty acid biosynthesis are constructed based on our medium confident bins. Each of the five circles in different colors encloses the corresponding one of the five interactive modules in the fatty acid biosynthesis depicted in Fig 3 (C). B: The sub-networks related to leucine catabolism. The colored circles represent the function modules corresponding to the function unit in Fig 3 (D). C: The fatty acid biosynthesis with the candidate genes in *Arabidopsis thaliana,* proteins in each square with one of the five different colors in the figure are the same as the proteins in the related sub-network in Fig 3(A). D: The leucine degradation pathway in mitochondrion, the proteins in different colored squares are the same proteins depicted in the corresponding sub-networks in Fig 3(B).

Up to now, several published papers have studied the function modules or metabolic processes based on experimental generated PPI sets. Wurtele ES and his colleagues have articulated several core metabolic processes in Arabidopsis. We collected the proteins in fatty acid biosynthesis and leucine degradation pathways and searched their first level neighbors in our high and medium confident network to extract the PPI subnetwork out (Figure 3 A, B). Several separate modules of PPI related to certain metabolic process were detected during the process in our network and are consistence with the protein groups contained in their pathways (Figure 3 C,D)[38]. It is found that the components of our PPI module are all assembled together in the metabolic process and almost have the same function validated by their research results. Furthermore, in their result (where the result comes from?), six genes have been detected highly correlated with the gene At4g34030 (Table 3). Three of them were also predicted in our low confident PPI network, but only one (At1g03090) was detected in the medium confident network. This result, to certain extent, indicates that the accuracy of PPI prediction is enhanced while the confidence of our PPI network increases.

**Table 3:** Genes have the highest correlation with the At4g34030 across 965 Arabidopsis ATH1 array chips

Corr.	Gene ID	LR in our interactome
0.9	At1g03090	580.206

0.84	At3g06850	None in our interactome

0.84	At3g13450	None in our interactome

0.82	At2g43400	0.7408

0.8	At3g45300	None in our interactome

0.8	At4g35770	0.7408

The genes with correlation greater than 0.8 across 965 chips, data from [38]

Furthermore, we carried out text mining for those newly published papers after our prediction was finished, eighty newly biological experiment validated PPI pairs was collected. Forty-eight of them can be converted to AGI code and mapped to our PPI network, thirty-four and six of them can be found in our medium and high confident PPI network, respectively (See additional file 5). The number of PPI pairs newly confirmed by other groups existed in both of our medium and high confident PPI network indicate the accuracy of our PPI prediction. Considering the coverage of medium confident interactome, we suggest researchers, who are interested our dataset, to pay more attention to our medium confident PPI network.

### Protein function prediction

**Figure 4:**
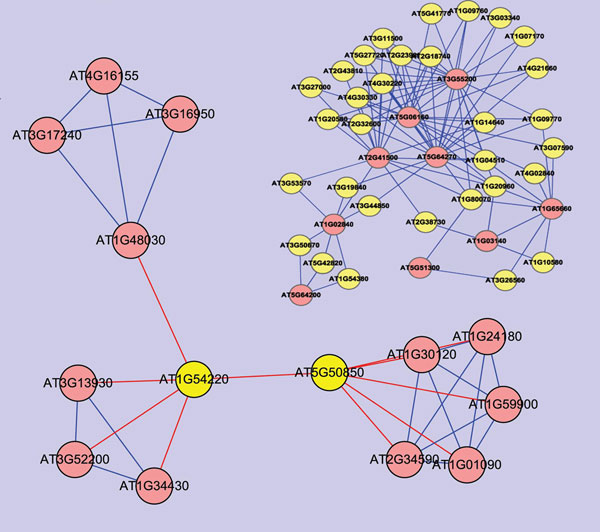
The sub-network related to ath00010 pathway shows the functional consistence The red line between At5g50850 and At1g54220 indicates the similarity of their functions. Other red lines indicate the same functions between two proteins. The relationship between At5g50850 and At1g54220 in our interactome proves their functional linkage.

The network view of the interactive proteins can be used to infer the potential new relationships between proteins. The basic underlying assumption of the functional annotation is that pairwise interaction is a strong indication for common function. Therefore, the PPI network offers a new resource for protein function study. We have used the dataset to annotate the unknown protein functions based on the theory of ‘guilty by association’ [15,16]. In our medium confident network, the protein At5g50850, which is annotated as pyruvate dehydrogenase, has an interaction with At1g54220, which is annotated as putative dihydrolipoamide S-acetyltransferase (Figure 4). As dihydrolipoamide S-acetyltransferase is generally accepted as the E2 component of pyruvate dehydrogenase complex, it is further suggested that the protein At1g54220 have the function of dihydrolipoamide S-acetyltransferase based on our data. Since the protein At5g50850 has been annotated with E.C 1.2.4.1 and has an action on 6-S-Acetyl-dihydrolipoamide in the pathway ath00010 graph from KEGG, the protein At5g50850 could also play the same role as dihydrolipoamide S-acetyltransferase. Moreover, other proteins that interact with At5g50850 are all annotated as pyruvate dehydrogenase and proteins that are connected with At1g54220 all have the annotation as a dihydrolipoamide S-acetyltransferase. These interactions further demonstrate that the function similarity exists between interactive protein pair. These evidences confirm the feasibility to predict protein function on the basis of PPI networks.

As discussed above, our interactome contains large amount of validated functional linkage information, which provide rich resource to investigate the potential new mechanisms and characterize the gene/protein new function under systematical level. Although our current interactome and other predicted protein interaction datasets for *Arabidopis* have certain limitation, they provide the first step towards understanding the architecture and function of the cellular network. It can be expected that the genome-wide protein interactome will be better delineated both in coverage and accuracy through the bioinformatics integration with the increase of the genomic and proteomic experimental data in the future.

## Conclusion

A predictive protein-protein interaction network in the organism plant *Arabidopsis thaliana* has been already constructed according to our research. Our interactome is a comprehensive genome-wide network and provides a rich resource for researchers in related field to study the protein function, molecular interaction and potential mechanism under different conditions. Anyone who are interested in the field of genome-wide protein-protein interaction network and corresponding annotation information could go to AtPID to access the comprehensive data.

## Methods

### A gold standard positive set and a gold standard negative set

A gold standard positive set (GSP) was constructed on the basis of the data from databases IntAct, BIND, TAIR and text mining. During the process of text mining, we have collected PPI data with experimental evidence and references. To ensure the reliability of these data, we also conducted a validation process. First, PPIs collected from the literature without AGI locus identifiers were mapped to IPI. Symbols without a match were removed. 3,866 protein-protein interaction pairs involving 1,875 proteins were extracted based on this filtration process. Additionally, protein components in partial enzyme complexes were also added to GSP based on the assumption that components of an enzyme complex have not only high functional association but also potential physical interactions. Enzyme complexes from KEGG[21] were downloaded, the overlapping part between these complex data and interaction data from text mining were extracted as a part of gold standard positive set. Subsequently we examined the property of PPI in protein complex. Because many subunits or components of an enzyme complex are constructed with regards to sequence similarity with other species or orthologs, validated physical protein interaction data was referred to reduce noise and redundant information here. By means of comparing the proteins pairs in complex with the existed part of GSP, we found that most protein-protein pairs of enzyme complex that had more than eighteen components did not have the evidence of interaction in our GSP. On the contrary, almost all protein pairs in the complex that processed less than eighteen components were supported by our GSP to have interactions. So we only added the protein pairs that existed in less than eighteen components complex into our GSP set. In all, 800 unique pairs were obtained according to enzyme complex after excluding the redundancies from the 3,866 pairs via text mining. Consequently, a total of 4,129 interaction pairs involving 2,285 proteins were collected to form a gold standard set. A gold standard negative set (GSN), contains 196,855 protein pairs, was set up based on the different sublocation information according to the Gene Ontology database. Only the data marked with evidence code of "IC", "IDA" and "IPI", that guaranteed high quality of the data, were taken into consideration in order to build gold standard negative set. 13 pairs of identical protein-pair, which appeared in both sets, were removed both from GSP and GSN. The LR score of any protein protein interaction pairs are calculated on the basis of our reliable GSP and GSN.

### Method of PPI prediction

In terms of the method of ortholog interactome, we followed the methods proposed by Chinnaiyan [4]. Firstly, we queried high-throughput interactome data in four model organisms: *Sacchromyces cerevisiae, Caenorhabditis elegans, Drosophila melanogaster* and *homo sapiens*[39-42]Secondly, we searched the ortholog proteins in *Arabidopsis thaliana* with the help of Inparanoid [43-45]. Then, four Arabidopsis interactomes were generated based on the four species. Each interactome has a LR score of its own. The data that presents the amount of predictive protein protein interaction pairs in *Arabidopsis thaliana* derived from the interactomes in the above four species is showed in table 1 and the final LR score used in Bayesian integrative approach is the largest LR generated with ortholog interactome among these four model organisms. (Additional file 6 table 1)

Gene expression profile is also generally used to infer protein protein interaction since previous studies have already demonstrate that the genes encoding interactive proteins have the similar expression patterns under different conditions. We have chosen five groups of chips: ME00345, ME00338, ME00331, ME00326 and ME00319 to detect the similarity of the gene expression patterns. After preprocessing of these chips with MAS5 we calculated the Pearson Correlation for each pair of genes among the above chips with R package[46]. The identified coexpressed proteins with their Pearson Correlation as LR have been integrated into our whole networks. (Additional file 6 table 4-8)

Domain is a function unit of a protein and participates in intermolecular interactions, each protein is a collection of conserved domains. Therefore, it can infer the protein interaction from related domain interaction, we could conclude that two proteins have interactions if domains in each protein have confirmed interactions. We got domain interactive information from Pfam database and induced the PPI information through mapping domain interactive data to protein information from TAIR (7). (Additional file 6 table 2)

Gene Ontology, that has a hierarchical structure, describes the biological relationship for both genes and its products of different species. A reasonable hypothesis is that the more components a GO term has, the less likely that interaction will exits between the components of the same GO term. We used the method of SSBP [4] to predict PPI on the structure of GO. We parsed the obo file from Gene ontology, and then counted the number of genes under each GO term. Next, we used the decision tree J48 to discrete SSBP score and calculate the LR. (Additional file 6 table 3)

Gene fusion, phylogenetic profile and gene neighbor were all exerted according to sequence information. Gene fusion method was based on the hypothesis that the homologues of two interactive proteins in one species may fuse into a single protein in another species. Phylogenetic profile supposed that two proteins with the similar profiles in different species might have interactions or functional linkages. The hypothesis of gene neighbor method is that proteins with neighborhood relationships would have a higher probability to have interaction or form a protein complex than the proteins that do not have such relationship. We also used these three genome context methods to infer the protein protein interactions in *Arabidopsis thaliana.* The result of these three methods were integrated with the results of domain and SSBP to form a new combined method to represent functional linkage and sequence similarity between two proteins, thus predicting the interactions between the two proteins. (Additional file 6 table 9-11)

### Data integration: Naïve Bayesian approach

This approach provides a mathematical rule to explain how to adjust the odds of a protein-protein pair interaction based on new predictive evidence. The prior odds of interactions can be calculated by the probability of finding an interacting pair among all protein pairs divided by the probability of finding a non-interacting pair.

O_prior_=P(pos)/P(neg)

The posterior odds or the odds that two proteins have interaction based on new predictive evidence are defined as:

Where  is a protein pair’s value in dataset i.

Likelihood score, which reflect both the sensitivity and specificity, for each corresponding set of prior odds were computed based on a derivation of Bayesian rule:

With the approach described above we could get the LR score of each PPI pair, and the main LR of a certain PPI pair is the product of the LR score from different methods.

The Naïve Bayesian method is used to integrate the datasets that are independent to each other. However, the independence of our methods of predicting PPI is hard to affirm and we are unable to prove that the features used in our study to be independent. We have explored the independence of these methods and calculated the combined LR of each two methods, but the criterion of independence between two methods is hard to be fixed (data not shown). Given that gene neighbor, gene fusion and phylogenetic profile are all predictive methods on the basis of the sequence information, their independence can therefore be regarded weakly. Furthermore, SSBP represents the functional consistence of two proteins more than real physical interaction and two proteins that have domain interactions may not have physical interactions because of protein location or other biological constrains. In order to follow the requirement of Bayesian approach and enhance the reliability of our predictive interactome, we combined the methods of gene neighbor, gene fusion, phylogenetic profile and SSBP together and validate the PPI pair obtained by these four methods with protein domain method. The result of this combined new PPI predictive method is treated as representing the aspect of functional consistent of a PPI pair, which is a new feature used in our Bayesian Integration process. As to the methods of ortholog and gene coexpression, their hypotheses based on which that a PPI pair has been predicted to have interaction are different and do not relate to the functional consistent. We have regarded that these three methods, including ortholog interactome, gene expression and the new combined method are independent to each other. The final LR score of our PPI network is the product of the LR scores of the three above methods.

## Competing interests

The authors do not consult to the supporter of this study. The authors declare that they have no competing interests.

## Authors' contributions

TS, YL designed the study and GL implemented the algorithms and data integration. GL and FX analyzed the results, FX and TS drafted the manuscript, FX, CZ, PL, JC and YD collected the data and participated in the analysis and discussion. All authors read and approved the final manuscript.

## Supplementary Material

Additional file 1 The annotation result of unknown proteins with annealing simulation algorithm on the basis of our interactome. Click here for file

Additional file 2 The conservative PPI are listed. Moreover, further information about the PPI pairs such as their ID, GO annotation, species , the experiment that validate the PPI, the related literature etc. are listed under the conservative PPI pairs. Click here for file

Additional file 3 The pathway related interactions are stored in additional file 5. The interactive pairs pertaining to the pathways are listed under each pathway name. Click here for file

Additional file 4 The first line of each part is the GO term that enriched in the tissue specific protein list. The information below the first line is the GO terms that enriched in the interactions partner of the first line listed GO term. All of the GO enrichment analysis related information including GO-ID, p-value, corr, total Description Genes in test set are listed. Click here for file

Additional file 5 This file contains the result of new published validated protein interaction pairs in our predictive interactome. Click here for file

Additional file 6 The tables in the file contains the information about the predictive result of the seven methods applied in our study. Click here for file
