# Supplementary material for: Global protein interactome exploration through mining genome-scale data in Arabidopsis thaliana
Source: BMC Genomics. 2010 Nov 2;11(Suppl 2):S2. doi: 10.1186/1471-2164-11-S2-S2 (PMC2975419; doi:10.1186/1471-2164-11-S2-S2)
Supplement: Additional file 2 — The conservative PPI are listed. Moreover, further information about the PPI pairs such as their ID, GO annotation, species , the experiment that validate the PPI, the related literature etc. are listed under the conservative PPI pairs. [file 1471-2164-11-S2-S2-S2.doc]

**Additional file 2**

Each table below contains the overlap between the predictive result and GSN (golden standard negative), GSP (golden standard positive), including their LR and other related information.

In the following tables $p_{\text{possible}}$is the number of possible GSP pairs, $n_{\text{possible}}$ is the possible GSN pairs, Total is the result of PPI prediction; LR and the corresponding probability is calculated using the following formula:

$$LR=\frac{\Pr(\text{Class|}p_{\text{possible}})}{\text{Pr(Class|}n_{\text{possible}}\text{)}}=\frac{\frac{Total\cap GSP}{p_{\text{possible}}}}{\frac{Total\cap GSN}{n_{\text{possible}}}}$$

The class in the equation will be substituted by different method during the LR calculation after predicting PPI pairs.

Table 1. Result of ortholog interactome

| speices | GSP | $p_{\text{possible}}$ | GSN | $n_{\text{possible}}$ | Total |  |  | LR |
| --- | --- | --- | --- | --- | --- | --- | --- | --- |
| *homo sapiens* | 60 | 126 | 1 | 572 | 1127 | 0.476190476190476 | 0.00174825174825175 | 272.38 |
| *Caenorhabditis elegans* | 5 | 18 | 0 | 100 | 411 | 0.277777777777778 | 0 | -- |
| *Sacchromyces cerevisiae* | 84 | 276 | 9 | 2742 | 15913 | 0.304347826086957 | 0.00328227571115974 | 92.72 |
| *Drosophila melanogaster* | 18 | 128 | 1 | 1203 | 2201 | 0.140625 | 0.000831255195344971 | 169.17 |

Table 2 Result of domain method

| GSP | $p_{\text{possible}}$ | GSN | $n_{\text{possible}}$ | Total |  |  | LR |
| --- | --- | --- | --- | --- | --- | --- | --- |
| 755 | 2061 | 1081 | 28285 | 570925 | 0.366327025715672 | 0.0382181368216369 | 9.59 |

Table 3. Result of Gene ontology method (SSBP)

| SSBP | GSP | GSN | TOTAL |  |  | LR |
| --- | --- | --- | --- | --- | --- | --- |
| total | 319 | 12401 | 789396 | 0.873972603 | 1 | 0.873972603 |
| (0,7] | 44 | 11 | 1319 | 0.120547945 | 0.000887025 | 135.9013699 |
| (7,22] | 19 | 62 | 4982 | 0.052054795 | 0.004999597 | 10.4117985 |
| (22,100000] | 256 | 12328 | 783095 | 0.701369863 | 0.994113378 | 0.70552301 |
| possible | 365 | 12401 |  |  |  |  |

Table 4. Result based on gene expression profile (chip: ME00319)

| Class | GSP | GSN | TOTAL | Pr(D|GSP) | Pr(D|GSN) | LR |
| --- | --- | --- | --- | --- | --- | --- |
| -1 |  |  | 20 | 0 | 0 | -- |
| -0.9 |  | 108 | 24970 | 0 | 0.000466 | 0 |
| -0.8 | 4 | 1300 | 517946 | 0.000747 | 0.005607 | 0.133246 |
| -0.7 | 34 | 4432 | 2639234 | 0.00635 | 0.019115 | 0.332212 |
| -0.6 | 84 | 11176 | 6823172 | 0.015689 | 0.048203 | 0.325484 |
| -0.5 | 158 | 18044 | 12069202 | 0.029511 | 0.077825 | 0.379193 |
| -0.4 | 238 | 23192 | 17291294 | 0.044453 | 0.100028 | 0.444401 |
| -0.3 | 372 | 26514 | 21644018 | 0.069481 | 0.114356 | 0.607581 |
| -0.2 | 430 | 25740 | 23800604 | 0.080314 | 0.111018 | 0.723429 |
| -0.1 | 454 | 22318 | 23299320 | 0.084796 | 0.096259 | 0.880921 |
| 0 | 450 | 19978 | 21069036 | 0.084049 | 0.086166 | 0.975431 |
| 0.1 | 504 | 17776 | 18333892 | 0.094135 | 0.076669 | 1.227814 |
| 0.2 | 406 | 16052 | 15580108 | 0.075831 | 0.069233 | 1.0953 |
| 0.3 | 448 | 13622 | 13046334 | 0.083676 | 0.058752 | 1.424208 |
| 0.4 | 408 | 11714 | 10701570 | 0.076205 | 0.050523 | 1.508312 |
| 0.5 | 396 | 8646 | 8553114 | 0.073963 | 0.037291 | 1.983427 |
| 0.6 | 314 | 5666 | 6641980 | 0.058648 | 0.024438 | 2.399879 |
| 0.7 | 280 | 3548 | 4796878 | 0.052297 | 0.015303 | 3.417517 |
| 0.8 | 226 | 1608 | 2826328 | 0.042211 | 0.006935 | 6.086374 |
| 0.9 | 148 | 420 | 750510 | 0.027643 | 0.001811 | 15.25979 |
| possible | 5354 | 231854 | 21040953 |  |  |  |

Table 5. Result based on gene expression profile (chip: ME00326)

| Class | GSP | GSN | TOTAL | Pr(D|GSP) | Pr(D|GSN) | LR |
| --- | --- | --- | --- | --- | --- | --- |
| -1 | 100 | 10456 | 4687932 | 0.017953 | 0.040748 | 0.440591 |
| -0.9 | 182 | 16908 | 11174420 | 0.032675 | 0.065892 | 0.495885 |
| -0.8 | 296 | 16824 | 12098538 | 0.053142 | 0.065565 | 0.81052 |
| -0.7 | 226 | 15382 | 12116544 | 0.040575 | 0.059945 | 0.676857 |
| -0.6 | 200 | 12610 | 12295848 | 0.035907 | 0.049143 | 0.730662 |
| -0.5 | 212 | 12610 | 12564338 | 0.038061 | 0.049143 | 0.774501 |
| -0.4 | 212 | 10872 | 12896988 | 0.038061 | 0.042369 | 0.898313 |
| -0.3 | 210 | 11030 | 13220942 | 0.037702 | 0.042985 | 0.877092 |
| -0.2 | 218 | 12052 | 13482010 | 0.039138 | 0.046968 | 0.833295 |
| -0.1 | 226 | 12380 | 14420052 | 0.040575 | 0.048246 | 0.840987 |
| 0 | 232 | 11290 | 13813660 | 0.041652 | 0.043998 | 0.946663 |
| 0.1 | 254 | 10848 | 13728940 | 0.045601 | 0.042276 | 1.078662 |
| 0.2 | 278 | 11604 | 13538178 | 0.04991 | 0.045222 | 1.103668 |
| 0.3 | 312 | 12884 | 13439736 | 0.056014 | 0.05021 | 1.115592 |
| 0.4 | 344 | 12792 | 13436528 | 0.061759 | 0.049852 | 1.238858 |
| 0.5 | 332 | 13284 | 13652528 | 0.059605 | 0.051769 | 1.151359 |
| 0.6 | 390 | 14956 | 14108028 | 0.070018 | 0.058285 | 1.201298 |
| 0.7 | 446 | 15848 | 14610916 | 0.080072 | 0.061761 | 1.296468 |
| 0.8 | 510 | 15734 | 13442410 | 0.091562 | 0.061317 | 1.49325 |
| 0.9 | 390 | 6236 | 5522186 | 0.070018 | 0.024302 | 2.881111 |
| possible | 5570 | 256600 | 2.48E+08 |  |  |  |

Table 6. Result based on gene expression profile (chip: ME00331)

| Class | GSP | GSN | TOTAL | Pr(D|GSP) | Pr(D|GSN) | LR |
| --- | --- | --- | --- | --- | --- | --- |
| -1 | 8 | 92 | 103886 | 0.001761 | 0.000435 | 4.047058 |
| -0.9 | 22 | 1400 | 1060584 | 0.004844 | 0.006623 | 0.731361 |
| -0.8 | 54 | 4142 | 3266442 | 0.011889 | 0.019594 | 0.606766 |
| -0.7 | 144 | 8084 | 5982402 | 0.031704 | 0.038242 | 0.829036 |
| -0.6 | 174 | 11852 | 8613558 | 0.038309 | 0.056067 | 0.683274 |
| -0.5 | 194 | 13566 | 10808476 | 0.042712 | 0.064175 | 0.66556 |
| -0.4 | 272 | 14724 | 12433726 | 0.059886 | 0.069653 | 0.859766 |
| -0.3 | 282 | 15396 | 13451046 | 0.062087 | 0.072832 | 0.852469 |
| -0.2 | 288 | 15956 | 13978868 | 0.063408 | 0.075481 | 0.840051 |
| -0.1 | 308 | 16378 | 14116648 | 0.067812 | 0.077478 | 0.87524 |
| 0 | 332 | 15450 | 13918252 | 0.073096 | 0.073088 | 1.000108 |
| 0.1 | 336 | 14572 | 13453462 | 0.073976 | 0.068934 | 1.073143 |
| 0.2 | 360 | 14446 | 12729510 | 0.07926 | 0.068338 | 1.159824 |
| 0.3 | 356 | 13724 | 11797186 | 0.07838 | 0.064923 | 1.207276 |
| 0.4 | 300 | 12794 | 10623194 | 0.06605 | 0.060523 | 1.09132 |
| 0.5 | 326 | 11738 | 9184504 | 0.071775 | 0.055528 | 1.29259 |
| 0.6 | 266 | 11118 | 7460132 | 0.058565 | 0.052595 | 1.113505 |
| 0.7 | 200 | 9080 | 5420816 | 0.044033 | 0.042954 | 1.025136 |
| 0.8 | 196 | 5722 | 3039758 | 0.043153 | 0.027068 | 1.59421 |
| 0.9 | 124 | 1156 | 700734 | 0.027301 | 0.005469 | 4.992306 |
| possible | 4542 | 211390 | 1.72E+08 |  |  |  |

Table 7. Result based on gene expression profile (chip: ME00338)

| class | GSP | GSN | TOTAL | Pr(D|GSP) | Pr(D|GSN) | LR |
| --- | --- | --- | --- | --- | --- | --- |
| -1 | 100 | 10310 | 4313312 | 0.01796 | 0.040818 | 0.439992 |
| -0.9 | 174 | 19048 | 10744026 | 0.03125 | 0.075413 | 0.414384 |
| -0.8 | 232 | 17594 | 11560714 | 0.041667 | 0.069657 | 0.598173 |
| -0.7 | 234 | 13826 | 11597648 | 0.042026 | 0.054739 | 0.767755 |
| -0.6 | 246 | 11628 | 11830514 | 0.044181 | 0.046037 | 0.959695 |
| -0.5 | 232 | 11342 | 12233894 | 0.041667 | 0.044904 | 0.927901 |
| -0.4 | 238 | 11034 | 12783096 | 0.042744 | 0.043685 | 0.978469 |
| -0.3 | 212 | 11960 | 13322024 | 0.038075 | 0.047351 | 0.804096 |
| -0.2 | 188 | 12172 | 13663042 | 0.033764 | 0.04819 | 0.700647 |
| -0.1 | 208 | 11562 | 14539926 | 0.037356 | 0.045775 | 0.816082 |
| 0 | 224 | 11342 | 14057874 | 0.04023 | 0.044904 | 0.895904 |
| 0.1 | 260 | 12008 | 13996158 | 0.046695 | 0.047541 | 0.982213 |
| 0.2 | 280 | 11900 | 13729776 | 0.050287 | 0.047113 | 1.067368 |
| 0.3 | 264 | 10946 | 13449400 | 0.047414 | 0.043336 | 1.094086 |
| 0.4 | 334 | 11086 | 13323784 | 0.059986 | 0.043891 | 1.366705 |
| 0.5 | 418 | 12332 | 13385832 | 0.075072 | 0.048824 | 1.537609 |
| 0.6 | 400 | 14992 | 13624374 | 0.071839 | 0.059355 | 1.210329 |
| 0.7 | 434 | 15852 | 13958626 | 0.077945 | 0.06276 | 1.241964 |
| 0.8 | 474 | 14674 | 13164664 | 0.085129 | 0.058096 | 1.465322 |
| 0.9 | 416 | 6974 | 5454616 | 0.074713 | 0.027611 | 2.705918 |
| possible | 5568 | 252582 | 2.45E+08 |  |  |  |

Table 8. Result based on gene expression profile (chip: ME00345)

| class | GSP | GSN | TOTAL | Pr(D|GSP) | Pr(D|GSN) | LR |
| --- | --- | --- | --- | --- | --- | --- |
| -1 | 4 | 1508 | 276740 | 0.000897 | 0.007324 | 0.122507 |
| -0.9 | 34 | 6046 | 1615034 | 0.007627 | 0.029365 | 0.259725 |
| -0.8 | 88 | 10018 | 3350434 | 0.01974 | 0.048656 | 0.4057 |
| -0.7 | 92 | 10912 | 5169912 | 0.020637 | 0.052998 | 0.389392 |
| -0.6 | 174 | 11482 | 7127988 | 0.039031 | 0.055767 | 0.699899 |
| -0.5 | 232 | 13566 | 9197532 | 0.052041 | 0.065888 | 0.789841 |
| -0.4 | 290 | 14356 | 11184450 | 0.065052 | 0.069725 | 0.932971 |
| -0.3 | 332 | 14558 | 12904644 | 0.074473 | 0.070706 | 1.053271 |
| -0.2 | 334 | 15464 | 14180294 | 0.074921 | 0.075107 | 0.997535 |
| -0.1 | 336 | 15060 | 14877054 | 0.07537 | 0.073144 | 1.030429 |
| 0 | 356 | 14740 | 14962318 | 0.079856 | 0.07159 | 1.115465 |
| 0.1 | 378 | 13724 | 14373458 | 0.084791 | 0.066656 | 1.272081 |
| 0.2 | 384 | 13380 | 13195022 | 0.086137 | 0.064985 | 1.325497 |
| 0.3 | 292 | 12216 | 11554902 | 0.0655 | 0.059332 | 1.10397 |
| 0.4 | 310 | 10740 | 9594082 | 0.069538 | 0.052163 | 1.333095 |
| 0.5 | 236 | 8400 | 7470176 | 0.052939 | 0.040798 | 1.297587 |
| 0.6 | 188 | 7090 | 5427196 | 0.042171 | 0.034435 | 1.224659 |
| 0.7 | 170 | 5850 | 3552600 | 0.038134 | 0.028413 | 1.342136 |
| 0.8 | 128 | 4802 | 1834352 | 0.028712 | 0.023323 | 1.231095 |
| 0.9 | 100 | 1982 | 419444 | 0.022432 | 0.009626 | 2.330236 |
| possible | 4458 | 205894 | 1.62E+08 |  |  |  |

Table 9. Result of gene fusion method

|  | GSP | GSN | TOTAL | Pr(CL|GSP) | Pr(CL|GSN) | LR |
| --- | --- | --- | --- | --- | --- | --- |
| predict | 74 | 54 | 312676 | 0.02377892 | 0.001369273 | 17.36609778 |
| possiple | 3112 | 39437 |  |  |  |  |

Table 10. Result of phylogenetic profile method

| Class | GSP | GSN | TOTAL | Pr(CL|GSP) | Pr(CL|GSN) | LR |
| --- | --- | --- | --- | --- | --- | --- |
| all | 244 | 123 | 102853 | 0.218051832 | 0.012673879 | 17.20482138 |
| [0,4.5e-05] | 239 | 66 | 65169 | 0.213583557 | 0.006800618 | 31.40649119 |
| [4.5e-05,1] | 5 | 57 | 37680 | 0.004468275 | 0.005873261 | 0.760782654 |
| possible | 1119 | 9705 |  |  |  |  |

Table 11. Result of gene neighbor method

| Class | GSP | GSN | TOTAL | Pr(CL|GSP) | Pr(CL|GSN) | LR |
| --- | --- | --- | --- | --- | --- | --- |
| all | 925 | 18 | 15778 | 0.549287411 | 0.000593687 | 925.2136118 |
| (-133,-62.486697] | 191 | 5 | 1036 | 0.113420428 | 0.000164913 | 687.7587886 |
| (-62.486697,10] | 734 | 13 | 14687 | 0.435866983 | 0.000428774 | 1016.54239 |
| possible | 1684 | 30319 |  |  |  |  |
